# Resuscitative Endovascular Balloon Occlusion of The Aorta (REBOA) And Mortality in Hemorrhagic Shock Associated with Severe Pelvic Fracture: a National Data Analysis

**DOI:** 10.1186/s12873-024-01020-y

**Published:** 2024-06-24

**Authors:** Nasim Ahmed, Yen-Hong Kuo

**Affiliations:** 1 Division of Trauma & Surgical Critical Care, Jersey Shore University Medical Center, Neptune, NJ USA; 2 Hackensack Meridian School of Medicine, Nutley, NJ USA; 3 Office of Research Administration, Hackensack Meridian Health Research Institute, Nutley, NJ USA; 4 Department of Medical Sciences, Hackensack Meridian School of Medicine, Nutley, NJ USA

**Keywords:** REBOA, Hemorrhagic shock, Severe pelvic fracture

## Abstract

**Background:**

The purpose of the study was to evaluate the mortality of patients who received Resuscitative Endovascular Balloon Occlusion of The Aorta (REBOA) in severe pelvic fracture with hemorrhagic shock.

**Methods:**

The American College of Surgeon Trauma Quality Improvement Program (ACS-TQIP) database for the calendar years 2017–2019 was accessed for the study. The study included all patients aged 15 years and older who sustained severe pelvic fractures, defined as an injury with an abbreviated injury scale (AIS) score of ≥ 3, and who presented with the lowest systolic blood pressure (SBP) of < 90 mmHg. Patients with severe brain injury were excluded from the study. Propensity score matching was used to compare the patients who received REBOA with similar characteristics to patients who did not receive REBOA.

**Results:**

Out of 3,186 patients who qualified for the study, 35(1.1%) patients received REBOA for an ongoing hemorrhagic shock with severe pelvic fracture. The propensity matching created 35 pairs of patients. The pair-matched analysis showed no significant differences between the group who received REBOA and the group that did not receive REBOA regarding patients’ demography, injury severity, severity of pelvic fractures, lowest blood pressure at initial assessment and laparotomies. There was no significant difference found between REBOA versus no REBOA group in overall in-hospital mortality (34.3% vs. 28.6, *P* = 0.789).

**Conclusion:**

Our study did not identify any mortality advantage in patients who received REBOA in hemorrhagic shock associated with severe pelvic fracture compared to a similar cohort of patients who did not receive REBOA. A larger sample size prospective study is needed to validate our results.

**Case–control retrospective study:**

Level of Evidence IV.

## Introduction

A severe pelvic fracture can be a life-threatening condition due to associated injuries. The overall mortality associated with pelvic fracture was reported as between 8–11% [1, 2]. Among patients who died due to pelvic fractures, 93% of them died due to hemorrhagic shock [3]. Over a few decades, numerous modalities have been explored to reduce the mortality that was attributed to hemorrhagic shock. Mass antishock trousers, pelvic binders, C-clamp, pelvic packing, and angioembolization are among many modalities introduced with some degree of success [4–13]. Intra-arterial balloon occlusion devices have also been used in the management of ongoing hemorrhage associated with pelvic fractures [14].

Resuscitative endovascular balloon occlusion of the aorta (REBOA) is a type of intra-arterial occlusion device that has been introduced in the management of trauma patients with ongoing hemorrhage from the torso. Prior studies on REBOA showed favorable mortality outcomes when the patient was compared to patients that had resuscitative thoracotomy (RT) [15–17]. Some of these studies also showed a better survival probability if the REBOA was placed in a hypotensive patient as compared to traumatic arrest. A recent randomized trial that was completed in United Kingdome on REBOA catheter placement ( in Zone I and Zone III) in trauma victims, however, the trial was stopped when 2nd interim analysis found a higher mortality in REBOA patients, and the prespecified stopping rule for harm was met [18]. This trial was designed to find the mortality outcomes of REBOA catheter use in trauma patient suspected for torso injury not specific to hemorrhagic shock associated with severe pelvic fracture. The use of REBOA in severe pelvic fractures with ongoing hemorrhage is evolving. Very few studies have examined the efficacy of the REBOA, as an adjunct to definite care, in controlling the hemorrhage associated with severe pelvic fracture. A recent study from the national trauma database of the US showed better outcomes with REBOA placement when compared with the patients with pre-peritoneal packing [19]. Another descriptive study from a level one trauma center in France included all patients with a severe pelvic fracture who underwent REBOA catheter as a bridge to definite hemorrhage control showed approximately 60% mortality, however, the study did not compare the REBOA patients with control patients [20]. A recent study from National Trauma Data Bank (NTDB) data set of 2016–2018 included all severe pelvic fractures with and without REBOA catheter placement showed higher mortality in REBOA group when compared to non-REBOA group [21]. Another recent study utilizing 2017 NTDB dataset that included all pelvic fractures with Abbreviated Injury Scale (AIS) > 1 with unstable hemodynamics found higher mortality in REBOA group when compared to Pelvic Angioembolization [22]. Both recent studies from NTDB data set have some concerns related to patient inclusion; one included severe head injury with head AIS of 3, another excluded severe head injury, however included all pelvic fracture with AIS > 1 with unstable hemodynamics. Both studies lack the detailed information of pelvic fracture. Therefore, this study was designed to evaluate the association of REBOA use with the mortality of patients with severe pelvic fractures excluding all severe head injury who presented with hemorrhagic shock using the national data. Our hypothesis is that REBOA will decrease the overall mortality in severe pelvic fractures with ongoing hemorrhage.

## Methods

**Fig. 1:**
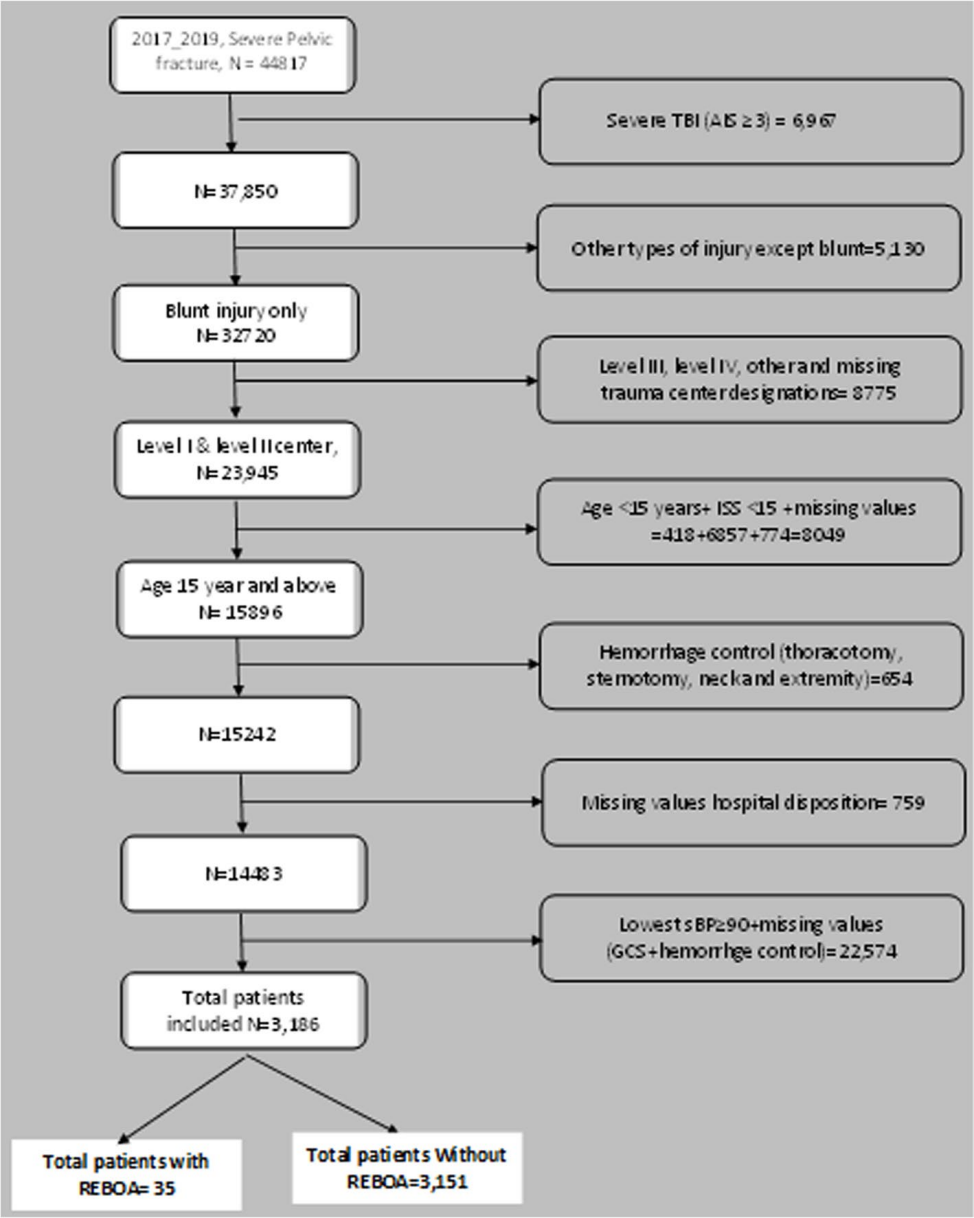
Flow Diagram showing the inclusion and exclusion of severe pelvic fracture patients with and without REBOA

The study was conducted using the Trauma quality improvement program (TQIP) Participant Use File (PUF) database, owned and maintained by the American College of Surgeons (ACS), for the calendar years 2017–2019. Individual trauma centers across the US participate in data sharing of injured patients with ACS on a voluntary basis. All the data entries in the trauma registry are entered by trauma registrars [13]. Included in the study were all severely injured ( injury severity score (ISS) > 15)) patients aged 15 years and older who sustained severe pelvic fractures, defined as an injury with an abbreviated injury scale (AIS) score of ≥ 3, with diagnosis code of pelvic fractures (856,101, 856,161,856,162,856,163,856,164, 856,171, 856,172, 856,173 and 856,174), who presented with lowest systolic blood pressure (SBP) of < 90 mmHg. The lowest sBP is defined as to lowest SBP in the ED/hospital of the index hospital, where index hospital is the hospital abstracting the data. Other variables included in the study were patients’ demography, ISS, Glasgow coma scale (GCS), and comorbidities; chronic alcoholism, use of anticoagulant, diabetes mellitus (DM), hypertension (HTN) on medication, and chronic pulmonary obstructive disease (COPD). Additional hemorrhage-controlled procedures for example, angiography and angioembolization, laparotomy, and pelvic packing were also included in the study. Patients who received blood transfusion (Packed Red Blood Cells [PRBC]) within the 4 h of patient arrival were also included in the study as were patients with severe injuries of different body regions with AIS ≥ 3. Patients with severe brain injury with AIS ≥ 3 or other forms of hemorrhage control procedure for example, thoracotomy, sternotomy, neck exploration, and exsanguination from the extremities were excluded from the study. Figure 1

The primary outcome was all-cause in-hospital mortality.

### Statistics

Summary statistics were performed using the median and interquartile range for the continuous variables and the number and percentage for the categorical variables. The two groups, who received REBOA (procedure code; 04L03DJ) and those who did not receive REBOA, were compared using the Wilcoxon Rank Sum test for continuous variables, and the Chi-square or Fisher exact test was used for the categorical variables. For the propensity score matching analysis, first calculated the group propensity score and matched with the patient who received REBOA with a subject who did not receive REBOA. MatchIt package from R was used to create the propensity score matching [23]. The characteristics used for calculating the propensity score were ( race [ white], sex, ISS, GCS, lowest SBP,). The improvement after matching was assessed with the numeric and graphical diagnostics. The Wilcoxon Signed Rank test, and McNemar's or the Stuart-Maxwell test were used to compare the two groups. The risk difference and odds ratio with their respective 95% confidence intervals were calculated. All *p*-values reported were 2-sided for each test. A *p*-value of < 0.05 was considered statistical significance. All analyses were performed using the R language [24].

## Results

### Univariate analysis

Out of 3,186 patients who qualified for the study, 35(1.1%) patients received REBOA for an ongoing hemorrhagic shock associated with severe pelvic fracture. For patients who received REBOA, the median age was 48 [IQR; 32–59], and they were predominantly male (80%) and Caucasian (80%). REBOA patients presented with significantly lower lowest SBP (63 mmHg [ IQR; 54.5–77.5] vs. 73 mmHg [IQR; 62–81], *P* = 0.005), and lower initial SBP (96 mmHg [IQR; 80 – 119] vs. 79.5 mmHg [IQR; 72 – 94], *P* = 0.001) and more frequently received higher number of PRBCs transfusion (median (IQR]; 13 [6 – 19.5] vs. 4 [2–8], *P* < 0.001), Angio-embolization (60% vs. 34.1%, *P* = 0.021) and laparotomy (62.9% vs. 37.2%, *P* = 0.003) compared to patients who did not receive REBOA. There were no significant differences found between the two groups regarding ISS, GCS, comorbidities, and different body regions injuries, except upper extremity.

#### Propensity matching analysis

After propensity matching, there was a 50% to 90% improvement in standardized mean differences in the majority of the selected variables. The propensity matching created 35 pairs of patients. The pair-matched analysis showed no significant differences between the group who received REBOA vs. did not receive REBOA regarding median age (48 [IQR; 32–59] vs. 53 [IQR; 31.5–59], *P* = 0.989]), race [white] (80% vs. 77.1%, *P* > 0.99), sex [male] (80% vs. 74.3%, *P* = 0.0.803), lowest SBP 63 mmHg [ IQR; 54.5–77.5] vs. 62 mmHg [ IQR; 54–72.5], *P* = 0.642) and angioembolization ( 60% vs. 37.1%, *P* = 0.131). The patients in the REBOA group received a higher number of units of PRBCs ( median [IQR]; 13 [6 – 19.5] vs. 4 [2 – 8.5] *P* = 0.006) and also had lower initial SBP(79.5 mmHg [IQR; 72 – 94] vs. 100 mmHg [IQR;82.3 – 125.5]*P* = 0.004); but, the same number of laparotomies (62.9% vs.62.9%) when compared to non-REBOA groups, respectively. No significant difference existed in other body injuries and comorbidities Table 1. The characteristics of the severity of pelvic fractures between the two groups were not significantly different Table 2.

**Table 1:** comparison of patients who received REBOA vs. No REBOA before and after propensity matching

		**Before Matching**			**After** **Matching**	
**Variable**	**No REBOA (*n* = 3151)**	**REBOA (*n* = 35)**	***P*-Value**	**No REBOA (*n* = 35)**	**REBOA (*n* = 35)**	***P*-Value**
Age (years), Median [Q1-Q3]	47 [ 30.5—61]	48 [ 32—59]	0.877	53 [ 31.5—59]	48 [ 32—59]	0.989
Race, n (%)						
American Indian	26 (0.8)	0 (0)	> 0.99	0 (0)	0 (0)	NA
Asian	78 (2.5)	1 (2.9)	> 0.99	0 (0)	1 (2.9)	NA
Black	448 (14.2)	3 (8.6)	0.467	4 (11.4)	3 (8.6)	> 0.99
Pacific-islander	14 (0.4)	0 (0)	> 0.99	0 (0)	0 (0)	NA
Race-other	307 (9.7)	2 (5.7)	0.574	1 (2.9)	2 (5.7)	> 0.99
white	2184 (69.3)	28 (80)	0.238	27 (77.1)	28 (80)	> 0.99
Sex, n (%)			0.172			0.803
Female	1017 (32.3)	7 (20)		9 (25.7)	7 (20)	
Male	2134 (67.7)	28 (80)		26 (74.3)	28 (80)	
ISS, Median [Q1-Q3]	33 [24—41]	34 [ 26.5—39.5]	0.799	33 [ 28—42.5]	34 [ 26.5—39.5]	0.395
GCS, Median [Q1-Q3]	14 [10–15]	14 [10–15]	0.29	15 [ 7.5—15]	14 [10–15]	0.613
Lowest SBP, Median [Q1-Q3]	73 [62—81]	63 [ 54.5—77.5]	0.005	62 [ 54—72.5]	63 [ 54.5—77.5]	0.642
Initial SBP, Median [Q1-Q3]^a^	96 [80 – 119]	79.5 [72 – 94]	0.001	100 [82.3 – 125.5]	79.5 [72 – 94]	0.004
Pelvic packing, n (%)	27 (0.9)	2 (5.7)	0.04	0 (0)	2 (5.7)	NA
RBCS transfusion (units) in 4 h	4 [2–8]	13 [6 – 19.5]	< 0.001	4 [2 – 8.5]	13 [6 – 19.5]	0.006
Angiography, n (%)			0.021			0.131
No angiogram	1801 (57.2)	12 (34.3)		20 (57.1)	12 (34.3)	
Angiogram only	265 (8.4)	2 (5.7)		2 (5.7)	2 (5.7)	
Angio-Embolization	1074 (34.1)	21 (60)		13 (37.1)	21 (60)	
Angio-stenting	11 (0.3)	0 (0)				
Laparotomy, n (%)	1173 (37.2)	22 (62.9)	0.003	22 (62.9)	22 (62.9)	NA
**Comorbidities, n (%)**						
Chronic alcoholism	213 (6.8)	3 (8.6)	0.512	4 (11.4)	3 (8.6)	> 0.99
Anticoagulant	128 (4.1)	2 (5.7)	0.652	2 (5.7)	2 (5.7)	> 0.99
Bleeding disorder	20 (0.6)	0 (0)	> 0.99	1 (2.9)	0 (0)	NA
Chemotherapy	6 (0.2)	0 (0)	> 0.99	0 (0)	0 (0)	NA
Cirrhosis	47 (1.5)	0 (0)	> 0.99	2 (5.7)	0 (0)	NA
COPD	110 (3.5)	1 (2.9)	> 0.99	1 (2.9)	1 (2.9)	> 0.99
CVA/Stroke	35 (1.1)	1 (2.9)	0.33	0 (0)	1 (2.9)	NA
Dementia	22 (0.7)	0 (0)	> 0.99	0 (0)	0 (0)	NA
Diabetes mellitus	289 (9.2)	1 (2.9)	0.367	6 (17.1)	1 (2.9)	0.131
Disseminated cancer	5 (0.2)	0 (0)	> 0.99	0 (0)	0 (0)	NA
Functional dependency	59 (1.9)	0 (0)	> 0.99	0 (0)	0 (0)	NA
CHF	40 (1.3)	0 (0)	> 0.99	1 (2.9)	0 (0)	NA
Hypertension	654 (20.8)	6 (17.1)	0.753	9 (25.7)	6 (17.1)	0.505
Chronic renal failure	16 (0.5)	1 (2.9)	0.172	0 (0)	1 (2.9)	NA
MI	8 (0.3)	0 (0)	> 0.99	0 (0)	0 (0)	NA
PAD	13 (0.4)	0 (0)	> 0.99	0 (0)	0 (0)	NA
Smoking	650 (20.6)	12 (34.3)	0.077	7 (20)	12 (34.3)	0.228
Steroid	12 (0.4)	0 (0)	> 0.99	0 (0)	0 (0)	NA
**Abbreviated Injury Scale (AIS) ≥ 3, n (%)**						
Neck	52 (1.7)	1 (2.9)	0.446	0 (0)	1 (2.9)	NA
Face	21 (0.7)	1 (2.9)	0.216	0 (0)	1 (2.9)	NA
Spine	159 (5)	2 (5.7)	0.696	1 (2.9)	2 (5.7)	> 0.99
Chest	1870 (59.3)	20 (57.1)	0.928	20 (57.1)	20 (57.1)	> 0.99
Abdomen	1614 (51.2)	20 (57.1)	0.598	20 (57.1)	20 (57.1)	> 0.99
Upper extremity	120 (3.8)	4 (11.4)	0.045	2 (5.7)	4 (11.4)	0.683
Lower extremity	1135 (36)	15 (42.9)	0.509	12 (34.3)	15 (42.9)	0.646

*ISS* Injury severity score, *GCS* Glasgow coma scale, *SBP* Systolic blood pressure < 90 mmHg, *COPD* Chronic obstructive pulmonary disease, *CVA* Cerebrovascular accident, *CHF* Congestive heart failure, *[Q1-Q3]* First quartile -3rd quartile, *n* number, % Percentage, *NA* Not applicable

^a^analysis were done before matching for initial sBP (3112 of no REBOA and 34 with REBOA), after matching (34 of each group REBOA)

**Table 2:** Characteristics of pelvic fractures between the groups after matching

	**No REBOA *N* = 35**	**REBOA *N* = 35**	***P*-values**
**Characteristics of Pelvic Fractures, n (%)**			**0.834**
Pelvic ring fracture NFS [includes pelvic ring dislocation], open	0 (0.0)	1(2.9)	
Pelvic ring fracture, complete disruption of posterior arch and disruption of the pelvic floor	2(5.7)	2(5.7)	
Pelvic ring fracture, complete disruption of posterior arch and disruption of the pelvic floor, blood loss > 20% by volume; large/extensive/expanding pelvic hematoma	2(5.7)	3(8.6)	
Pelvic ring fracture, complete disruption of posterior arch and disruption of the pelvic floor, open	1(2.9)	2(5.7)	
Pelvic ring fracture, complete disruption of posterior arch and pelvic floor NFS	3(8.6)	2(5.7)	
Pelvic ring fracture, complete disruption of posterior arch and pelvic floor, blood loss < = 20% by volume	2(5.7)	0(0.0)	
Pelvic ring fracture, complete disruption of posterior arch and pelvic floor, blood loss > 20% by volume	2(5.7)	3(8.6)	
Pelvic ring fracture, complete disruption of posterior arch and pelvic floor, open	0(0.0)	1(2.9)	
Pelvic ring fracture, incomplete disruption of posterior arch	4(11.4)	7(20.0)	
Pelvic ring fracture, incomplete disruption of posterior arch NFS	5(14.3)	4(11.4)	
Pelvic ring fracture, incomplete disruption of posterior arch, blood loss < = 20% by volume	3(8.6)	4(11.4)	
Pelvic ring fracture, incomplete disruption of posterior arch, blood loss < = 20% by volume; moderate pelvic hematoma	1(2.9)	1(2.9)	
Pelvic ring fracture, incomplete disruption of posterior arch, blood loss > 20% by volume	4(11.4)	3(8.6)	
Pelvic ring fracture, incomplete disruption of posterior arch, blood loss > 20% by volume; large/extensive/expanding pelvic hematoma	3(8.6)	1(2.9)	
Pelvic ring fracture, incomplete disruption of posterior arch, open	0(0.00)	1(2.9)	
Pelvic ring fracture, open, NFS	3(8.6)	0(0.0)	

### Outcomes

Higher mortality was associated with the REBOA group when compared with the no REBOA group (34.3% vs. 17.5%, *P* = 0.018) in univariate analysis. After propensity score matching, no significant difference was found between the two groups, REBOA vs. No REBOA, in overall in-hospital mortality (34.3% vs. 28.6%, *P* = 0.789). There were no significant differences found between the two groups in post-matching analysis regarding the hospital length of stay (Median (95% CI) [Kaplan–Meier procedure]; (22 [12, 44] vs. 8 [6, 17], *P* = 0.194) and ICU days ( median [IQR]; 9 [4 – 21.5] vs. 6 [3–18], *P* = 0.597) respectively Table 3.

**Table 3:** Mortality & length of stays between the groups with or without REBOA

Before matching	No REBOA (*n* = 3151)	REBOA (*n* = 35)		*P*-Value	Odds Ratio (95% CI)			Absolute Risk Difference (95% CI)
Mortality before matching, n (%)	552 (17.5)	12 (34.3)		0.018	2.457 [1.215, 4.967]			0.168 [0.111, 0.224]
hospital length of stay, Median (95% CI) [Kaplan–Meier procedure])	13 (12, 13)	22 (12, 44)		0.203				
ICU days, median [1st quartile -3rd quartile]	6 [3–13]	9 [4 – 21.5]		0.151				
**After Matching**	**No REBOA (*n* = 35)**	**REBOA (*n* = 35)**						
Mortality after matching, n (%)	10 (28.6)	12 (34.3)		0.789	1.333 [0.382, 6.483]			0.057 [-0.18, 0.294]
hospital length of stay, Median (95% CI) [Kaplan–Meier procedure])	8 (6, 17)	22 (12, 44)		0.194				
ICU days, median [1st quartile -3rd quartile]	6 [3–18]	9 [4 – 21.5]		0.597				
**Results from a Multiple Logistic Regression Model**								
**Variables**	**β coefficient**	**95% CI for β**			**OR**	**95% CI for OR**		***p* value**
(Intercept)	0.056	-0.567	0.679					0.8602
group	0.553	-0.218	1.324		1.738	0.804	3.758	0.1597
white	-0.163	-0.382	0.055		0.849	0.682	1.057	0.1426
sexMale	0.035	-0.188	0.259		1.036	0.828	1.296	0.7559
ISS	0.026	0.017	0.036		1.027	1.018	1.036	< .0001
GCS	-0.076	-0.097	-0.056		0.927	0.908	0.946	< .0001
lowest-sbp	-0.034	-0.04	-0.028		0.966	0.961	0.972	< .0001
laparotomy	0.665	0.441	0.889		1.944	1.554	2.432	< .0001
>= 5 Units of packed red blood cells (PRBC)	0.609	0.381	0.838		1.839	1.463	2.311	< .0001
**AISPREDOT Analysis**								
Abdomen								
**aispredot**	**No REBOA**	**(%)**	**REBOA**		**(%)**			
520,202	0	0.0	1		2.9			
520,206	0	0.0	1		2.9			
520,406	1	2.9	0		0.0			
520,602	0	0.0	1		2.9			
520,604	2	5.7	4		11.4			
520,606	0	0.0	1		2.9			
520,608	1	2.9	1		2.9			
520,699	1	2.9	3		8.6			
521,108	0	0.0	1		2.9			
521,206	1	2.9	0		0.0			
521,408	2	5.7	0		0.0			
540,624	0	0.0	1		2.9			
540,640	2	5.7	0		0.0			
541,426	1	2.9	1		2.9			
541,624	1	2.9	0		0.0			
541,626	2	5.7	0		0.0			
541,824	0	0.0	2		5.7			
541,826	2	5.7	1		2.9			
541,828	0	0.0	1		2.9			
544,226	0	0.0	1		2.9			
544,228	2	5.7	0		0.0			
544,240	2	5.7	0		0.0			
N/A	15	42.9	15		42.9			
**Total**	**35**		**35**					
**aispdescription**	**No REBOA**	**(%)**	**REBOA**		**(%)**			
Aorta, abdominal, intimal tear [includes dissection], no disruption	0	0.0	1		2.9			
Aorta, abdominal, laceration; perforation; puncture, minor; superficial; incomplete circumferential involvement; blood loss < = 20% by volume	0	0.0	1		2.9			
Bladder (urinary), laceration, extraperitoneal wall > 2 cm; intraperitoneal wall < = 2 cm [OIS III]	0	0.0	1		2.9			
Bladder (urinary), rupture	1	2.9	0		0.0			
Bladder (urinary), rupture NFS	1	2.9	0		0.0			
Celiac artery, laceration; perforation; puncture, minor; superficial; incomplete circumferential involvement; blood loss < = 20% by volume	1	2.9	0		0.0			
Iliac artery [common, internal, external] and its named branches [includes gluteal] NFS	0	0.0	2		5.7			
Iliac artery [common, internal, external] and its named branches [includes gluteal], laceration; perforation; puncture NFS	1	2.9	3		8.6			
Iliac artery [common, internal, external] and its named branches [includes gluteal], laceration; perforation; puncture, minor; superficial; incomplete circumferential involvement; blood loss < = 20% by volume	0	0.0	1		2.9			
Iliac artery [common, internal, external] and its named branches injury NFS	1	2.9	1		2.9			
Iliac artery [common, internal, external] and its named branches, intimal tear, no disruption	0	0.0	1		2.9			
Iliac artery [common, internal, external] and its named branches, laceration; perforation; puncture NFS	1	2.9	1		2.9			
Iliac artery [common, internal, external] and its named branches, laceration; perforation; puncture, major; rupture; transection; segmental loss; blood loss > 20% by volume	1	2.9	1		2.9			
Jejunum-ileum (small bowel), laceration, massive; avulsion; complex; tissue loss; transection; large areas of tissue devitalization or devascularization [OIS IV, V]	1	2.9	1		2.9			
Kidney, laceration, > 1 cm parenchymal depth of renal cortex, no collecting system rupture or urinary extravasation; moderate [OIS III]	1	2.9	0		0.0			
Kidney, laceration, parenchymal laceration extending through renal cortex, medulla and collecting system; main renal vessel injury with contained hemorrhage; major [OIS IV]	2	5.7	0		0.0			
Liver, laceration, > 3 cm parenchymal depth; major duct involvement; blood loss > 20% by volume; moderate [OIS III]	0	0.0	1		2.9			
Liver, laceration, > 3 cm parenchymal depth; major duct involvement; moderate [OIS III]	0	0.0	1		2.9			
Liver, laceration, parenchymal disruption < = 75% hepatic lobe; multiple lacerations > 3 cm deep; "burst" injury; major [OIS IV]	1	2.9	0		0.0			
Liver, laceration, parenchymal disruption < = 75% of hepatic lobe or < = 3 Couinard's segments within a single lobe; multiple lacerations > 3 cm deep; "burst" injury; major [OIS IV]	1	2.9	1		2.9			
Liver, laceration, parenchymal disruption of > 75% of hepatic lobe or > 3 CouinardÂ’s segments within a single lobe; or involving retrohepatic vena cava/central hepatic veins; massive; complex [OIS V]	0	0.0	1		2.9			
Other named arteries, abdomen, [e.g. hepatic, renal, splenic], laceration; perforation; puncture, major; rupture; transection; segmental loss; blood loss > 20% by volume	1	2.9	0		0.0			
Other named arteries, abdomen, [e.g., hepatic, renal, splenic], laceration; perforation; puncture, major; rupture; transection; segmental loss; blood loss > 20% by volume	1	2.9	0		0.0			
Spleen, laceration, hilar disruption producing total devascularization; tissue loss; avulsion; massive [OIS V]	1	2.9	0		0.0			
Spleen, laceration, hilar disruption producing total devascularization; tissue loss; avulsion; massive; completely shattered spleen [OIS V]	1	2.9	0		0.0			
Spleen, laceration, involving segmental or hilar vessels producing major devascularization of > 25% of spleen but no hilar injury; major (OIS IV)	0	0.0	1		2.9			
Spleen, rupture NFS	2	5.7	0		0.0			
Superior mesenteric artery, laceration; perforation; puncture, major; rupture; transection; segmental loss; blood loss > 20% by volume	0	0.0	1		2.9			
Vena Cava, inferior, laceration; perforation; puncture, major; rupture; transection; segmental loss; blood loss > 20% by volume	1	2.9	0		0.0			
N/A	15	42.9	15		42.9			
**Total**	**35**		**35**					

There were no significant differences found between the groups regarding in-hospital complication rates, including acute kidney injury, deep vein thrombosis, or extremity compartment syndrome Table 4.

**Table 4:** Comparison of Complications between the group's post-match analysis

Complications, n (%)	(*n* = 70))	No REBOA (*n* = 35)	REBOA (*n* = 35)	*P* Values
Superficial incision SSI	2 (2.9)	0 (0)	2 (5.7)	0.493
Deep SSI	1 (1.4)	1 (2.9)	0 (0)	> 0.999
Organ space SSI	1 (1.4)	1 (2.9)	0 (0)	> 0.999
Deep vein thrombosis	1 (1.4)	1 (2.9)	0 (0)	> 0.999
Cardiac arrest	2 (2.9)	0 (0)	2 (5.7)	0.493
CAUTI	2 (2.9)	1 (2.9)	1 (2.9)	> 0.999
Pulmonary embolism	2 (2.9)	2 (5.7)	0 (0)	0.493
Extremity compartment syndrome	3 (4.3)	0 (0)	3 (8.6)	0.239
Unplanned re-intubation	3 (4.3)	1 (2.9)	2 (5.7)	> 0.999
Acute kidney Injury	5 (7.1)	1 (2.9)	4 (11.4)	0.356
Other complications	4 (5.7)	2 (5.7)	2 (5.7)	> 0.999
ARDS	2 (2.9)	1 (2.9)	1 (2.9)	> 0.999
Unplanned return to the operating room	3 (4.3)	0 (0)	3 (8.6)	0.239
Sepsis	3 (4.3)	1 (2.9)	2 (5.7)	> 0.999
Stroke	1 (1.4)	1 (2.9)	0 (0)	> 0.999
Pressure ulcer	2 (2.9)	0 (0)	2 (5.7)	0.493
Unplanned ICU admission	1 (1.4)	1 (2.9)	0 (0)	> 0.999

*SSI* Surgical site infection, *CAUTI* Catheter-associated urinary tract infection, *ARDS* Acute respiratory distress syndrome

## Discussion

Our study showed only 1.1% of patients received REBOA for hemorrhagic shock associated with severe pelvic fracture. The mortality in the REBOA group was 34.3% compared to 28.6% in non REBOA group, *P* = 0.789.

We used propensity score matching to find the association of REBOA with overall in-hospital mortality. The propensity score matching has been described as a better methodology for observational study [25]. Our study showed that the mortality in REBOA patients who presented with hemorrhagic shock was 34.3% which is much less than the previous study in which an intra-aortic balloon occlusion device was used [14]. The study included 13 patients with the successful placement of an Intra-aortic balloon occlusion device in uncontrolled hemorrhage with pelvic fractures. Seven out of 13 patients (54%) died [14].

Many studies have shown favorable mortality outcomes in REBOA compared to RT [15–17]. However, a recent randomized trial from United Kingdom in patients who were suspected of torso injury showed a higher 90 day mortality (54% vs. 42%), Odds ratio (1.58 [95% credible interval, 0.72–3.52]) in REBOA group when compared to standard management group [18]. The trial initially planned to enroll 120 patients, but the trial was stopped due to prespecified rule of harm was met on 2nd interim analysis. Very limited data on REBOA use in severe pelvic fracture patients with ongoing hemorrhage are available. In 2015, Moore et al. reported the use of REBOA in 24 trauma patients and compared it with open resuscitative thoracotomies and found a better survival rate with REBOA [15]. In their study, REBOA was placed in 4 patients with unstable hemodynamics that were associated with pelvic fracture, 3 out of 4 patients survived. A study that collected data over the period of 20 years, from a level-one trauma center in France published a report of REBOA in hemorrhagic shock with severe pelvic fracture [20]. The study included 32 patients with the blunt mechanism of injury. Most of the REBOA patients, approximately 70%, sustained severe injuries with a median ISS of 44 due to motor vehicle crashes or falls. Overall 28 days mortality was 59% and the majority (55%) of them died within 24 h of arrival at the hospital. Another recent study from the TQIP database included all patients with severe pelvic fractures who presented with initial SBP < 100 mmHg [19]. The patients were divided into three groups, preperitoneal packing (PP), REBOA-only group, and REBOA + PP. The analysis showed the lowest in-hospital mortality in REBOA only group when a comparison was made among, the three groups [ PP vs. REBOA vs. REBOA + PP] (44% vs 29% vs 54%; *p* = 0.034). A meta-analysis on REBOA use in major exsanguination including rupture abdominal aortic aneurysm (AAA), traumatic injury, and other conditions, showed improvement in SBP after placement of the REBOA catheter. The review showed 63.0% (545/865) mortality in trauma patients who received REBOA which was significantly better than the trauma patients managed by alternative means [26]. A recent study utilizing 2016–2018 NTDB dataset including all pelvic fracture patients who underwent REBOA catheter placement found to have a higher odd of death (OR: 2.017, 95% CI: 1.065–3.819, *p* = 0.031). Another recent study using the 2017 NTDB dataset using all pelvic fracture with AIS score > 1, with unstable hemodynamics did not find any in-hospital mortality benefit with REBOA catheter placement (adjusted odds ratio [95% CI]: 1.45 [0.82–2.56]) [22].

Contrary to the above studies, our study evaluated the mortality in patients who presented with hemorrhagic shock with severe pelvic fracture with AIS score ≥ 3 and received REBOA and compared the patients with the same characteristics who did not receive REBOA and found no significant difference in mortality (34.3% vs. 28.6%, *P* = 0.789). Our results differed from a recently published similarly designed study showing higher mortality with REBOA [26]. One of the reasons for this difference in results would be our study specifically evaluated the patients with severe pelvic fractures instead of all trauma patients. Our mortality was lower (34.3% vs. 59%) than the study of REBOA in pelvic fracture from France [20]. The probable reason for lower mortality in our group may be a less severity of injury in our patients’ cohort with a median ISS at 33.5 compared to 44 in their report. Similarly, lower mortality (29% vs. 34.3%) was reported in the recent study on pelvic fracture when the REBOA-only group was compared with our study and the probable reason for lower mortality in their study may be the lower median ISS score at 28 when compared to 33.5 in our study [19]. Contrary to other study [27]. Our study showed higher number of PRBCs transfusion within 4 h in REBOA group compared to non-REBOA group. The exact reason for the higher blood transfusion amount in the REBOA group is not known. Consistent with previous study [28] our study did not show any difference in in-hospital complications.

### Limitations

This is a retrospective design study and carries some inherent limitations. We performed propensity score matching to reduce the selection bias. However, the propensity score does not consider unobserved variables. The other limitation of the data set is the unavailability of follow-up hemodynamic parameters of the initial resuscitation effort. Another limitation is the database does not provide expertise among the providers in different institutions. Furthermore, the study consisted of a small sample size. These factors may have impacted the results. Other limitations of the study were the lack of availability of hourly blood transfusion information and the use of ISS in our model instead of AIS score for creating the propensity matching analysis, which may have impacted the results.

## Conclusion

Although our study did not identify any mortality advantage in patients who received REBOA in hemorrhagic shock associated with severe pelvic fracture compared to a similar cohort of patients who did not receive REBOA, but due to small sample size a definite conclusion cannot be made.

## Data Availability

The data is available from the American College of Surgeons.
